# Supplementary material for: Oligodendrocytes express synaptic proteins that modulate myelin sheath formation
Source: Nat Commun. 2019 Sep 11;10:4125. doi: 10.1038/s41467-019-12059-y (PMC6739339; doi:10.1038/s41467-019-12059-y)
Supplement: Supplementary file 1 — Supplementary Information [file 41467_2019_12059_MOESM1_ESM.pdf]

Oligodendrocytes express synaptic proteins that modulate myelin sheath formation

Hughes and Appel

SUPPLEMENTARY MATERIAL

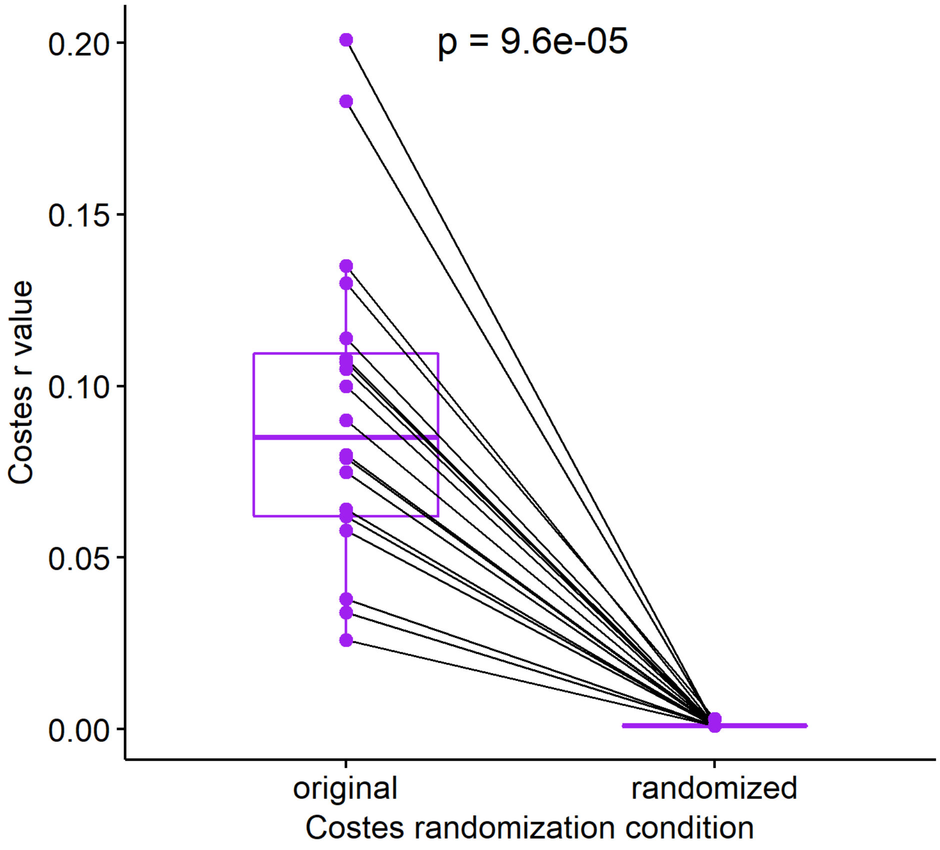

Supplementary Figure 1. Costes randomization analysis of PSD95 immunofluorescence colocalization with *Tg(mbpa:eGFP-CAAX)* fluorescence. Costes colocalization analysis of fluorescent signal between anti-PSD95 labeled with AF647 and transgenic reporter *Tg(mbpa:eGFP-CAAX)* before (original) or after (randomized) performing 1000 rounds of randomization per image for n=20 images containing both channels. Analysis was performed with the Fiji plugin JACoP with specific parameters: confocal image, bin width=0.001, pixel size=0.174 µm, randomizing in both the xy and z directions. Data are presented pairwise, analyzed by paired Wilcoxon, and the value plotted for the randomized condition is the maximum value from the output range (e.g., 0 +/- 0.003 is plotted as +0.003).

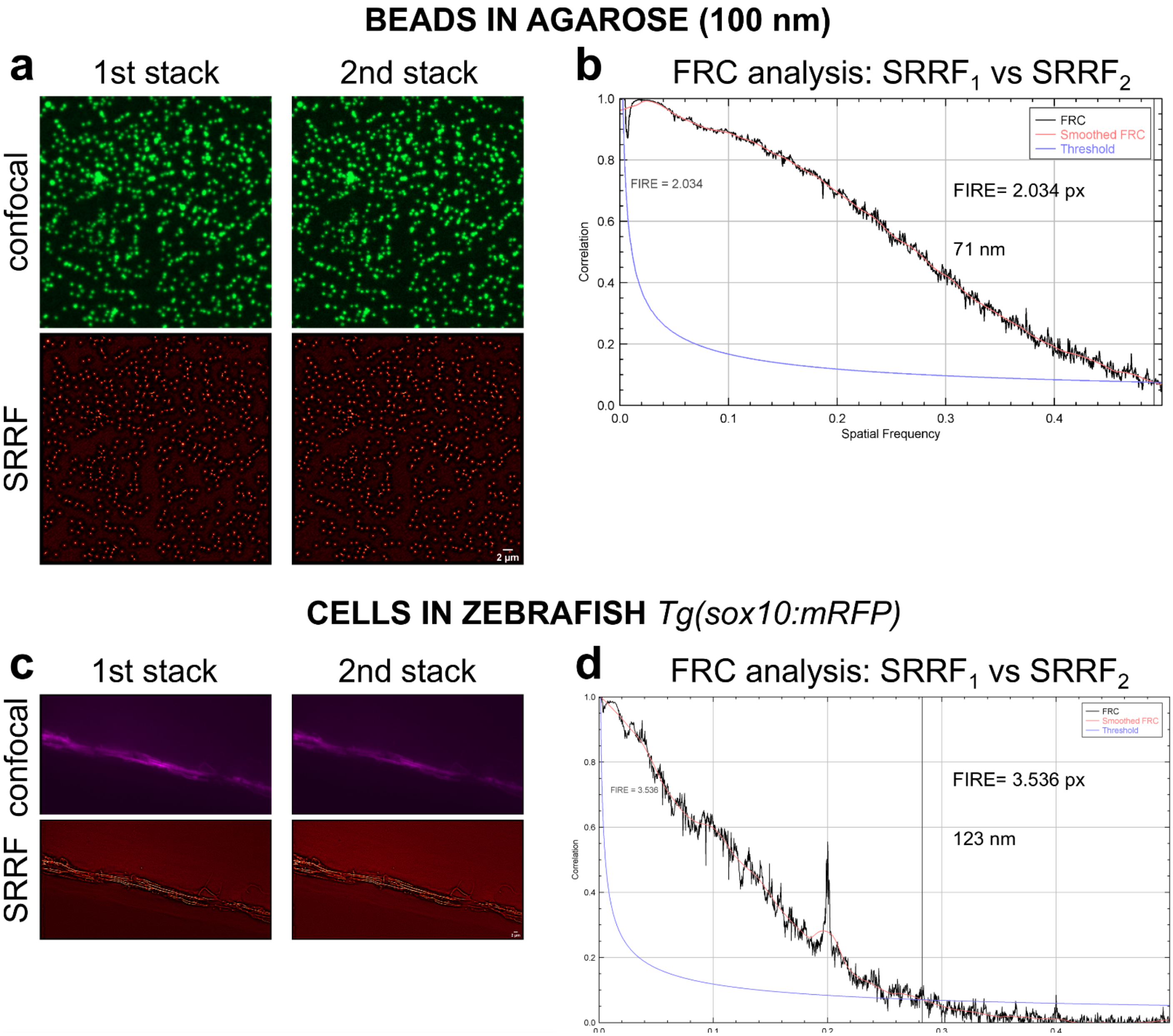

Supplementary Figure 2. Fourier ring correlation (FRC) analysis of resolution achieved by super-resolution radial fluctuation (SRRF) processing. (a) Single optical sections (75 frames) of fluorescent 100 nm beads suspended in agarose were acquired in two independent acquisitions (top) to generate SRRF reconstructions (bottom). (b) SRRF reconstructions were compared by 3-sigma FRC analysis to generate FRC curves and resolution values in both pixels and nanometers (71 nm resolution) (c) Single optical sections (109 frames) of oligodendrocytes in a live *Tg(sox10:mRFP)* agarose-mounted larva acquired twice and processed with SRRF. (d) SRRF reconstructions were compared by 3-sigma FRC analysis to generate FRC curves and resolution values in both pixels and nanometers (123 nm resolution). Scale bars, 2 µm.

Supplementary Table 1. Table of oligonucleotides

Construct	Template	Primer direction + flanks	Sequence
pME-vamp2	cDNA	F-attB1	GGG GAC AAG TTT GTA CAA AAA AGC AGG CTA CC ATGTCTGCCCCAGCCGG
		R-attB2	GGG GAC CAC TTT GTA CAA GAA AGC TGG GTT GGTGCTGAAGTACACAATGATTATAA
pME-cadm1b (wt)	cDNA	F-attB1	GGG GAC AAG TTT GTA CAA AAA AGC AGG CTA CC ATGAAAAGTCTGAAGCAGGTGT
		R-attB2	GGG GAC CAC TTT GTA CAA GAA AGC TGG GTT AATGTAGTATTCCTTCTTGTCATC
Ig1-dnCadm1b frag1 (N-term to Ig1)	cDNA	F	ATGAAAAGTCTGAAGCAGGTGT
		R-BamHI	CGC GGATCC CACTGAGACATTATTTGTCACCAG
Ig1-dnCadm1b frag2 (post-Ig1 to C-term)	cDNA	F-BamHI	CGC GGATCC GTTCCACCAGGCAACCCAATC
		R	AATGTAGTATTCCTTCTTGTCATC
pME-Ig1-dnCadm1b	Ig1-dnCadm1b frag1+2 ligation	F-attB1	GGG GAC AAG TTT GTA CAA AAA AGC AGG CTA CC ATGAAAAGTCTGAAGCAGGTGT
		R-attB2	GGG GAC CAC TTT GTA CAA GAA AGC TGG GTT AATGTAGTATTCCTTCTTGTCATC
pME-PSD95.FingR-GFP-CCR5TC-KRAB(A)	Addgene 72638	F-attB1	GGG GAC AAG TTT GTA CAA AAA AGC AGG CTA CC ATGCTCGAAGTCAAGGAAGC
		R-attB2	GGG GAC CAC TTT GTA CAA GAA AGC TGG GTT AGCCATAGAAGCAAGATTAGAATAA

pME-sypb	cDNA	F-attB1	GGG GAC AAG TTT GTA CAA AAA AGC AGG CTA CC ATGGATGTTGCCAACCAGTTGGTCG
		R-attB2	GGG GAC CAC TTT GTA CAA GAA AGC TGG GTT CATCTCGTTGGAGAAGGACGTGGG
p3E-cadm1b	cDNA	F-attB2r	GGG GAC AGC TTT CTT GTA CAA AGT GG AA ATGGCGATCTCGGGACTCGG
		R-attB3	GGG GAC AAC TTT GTA TAA TAA AGT TG AATGTAGTATTCCTTCTTGTCATCCG
pME-nlgn1 delPDZ (-STTRV)	SourceBioscience danio rerio nlgn1, cat# IMAGp998B1911381Q	F-attB1	GGG GAC AAG TTT GTA CAA AAA AGC AGG CTA CC ATGCCCTTCCAACAACCAAACC
		R-attB2	GGG GAC CAC TTT GTA CAA GAA AGC TGG GTT GTGTGAATGAGGGTGTGGATGGG
pME-nlgn2b delPDZ (-STTRV)	SourceBioscience danio rerio nlgn2b, cat# IMAGp998J1117607Q	F-attB1	GGG GAC AAG TTT GTA CAA AAA AGC AGG CTA CC ATGTCATCGGTGGACGTGGC
		R-attB2	GGG GAC CAC TTT GTA CAA GAA AGC TGG GTT GTGCTGATGGGGCAGGGC
pME-lrrtm2 delICD,delPDZ	cDNA	F-attB1	GGG GAC AAG TTT GTA CAA AAA AGC AGG CTA CC ATGGGTTTCCATTCAAGGTGGC
		R-attB2	GGG GAC CAC TTT GTA CAA GAA AGC TGG GTT GTTCTGAATGGCCGAGCAATGG
pME-lrrtm2 (wt full length)	cDNA	F-attB1	GGG GAC AAG TTT GTA CAA AAA AGC AGG CTA CC ATGGGTTTCCATTCAAGGTGGC
		R-attB2	GGG GAC CAC TTT GTA CAA GAA AGC TGG GTT TACTTCACACTCTTTGTAAGGCAG
pME-lrrtm1 delICD,delPDZ	cDNA	F-attB1	GGG GAC AAG TTT GTA CAA AAA AGC AGG CTA CC ATGCTAATGGATTTCCTTCTAATTGG

		R-attB2	GGG GAC CAC TTT GTA CAA GAA AGC TGG GTT CTGGCTGGTGAAGCACTGC
pME-lrrtm1 (wt full length)	cDNA	F-attB1	GGG GAC AAG TTT GTA CAA AAA AGC AGG CTA CC ATGCTAATGGATTTCCTTCTAATTGG
		R-attB2	GGG GAC CAC TTT GTA CAA GAA AGC TGG GTT CACTTCGCACTCTCGAGATGC
pME-lrrc4ba delPDZ (-ETQI)	SourceBioscience danio rerio lrrc4ba, cat# IMAGp998H1711205Q	F-attB1	GGG GAC AAG TTT GTA CAA AAA AGC AGG CTA CC ATGCGCATCACCACGGTGACC
		R-attB2	GGG GAC CAC TTT GTA CAA GAA AGC TGG GTT TTGGACATTCTCCTTGGAGCCAC
pME-cadm1b delPDZ (-KEYYI)	cDNA	F-attB1	GGG GAC AAG TTT GTA CAA AAA AGC AGG CTA CC ATGAAAAGTCTGAAGCAGGTGT
		R-attB2	GGG GAC CAC TTT GTA CAA GAA AGC TGG GTT CTTGTCATCCGAATTGTTGTGTCCG
pME-GCaMP6s-CAAX	p3E-2A-GCaMP6s-CAAX	F-attB1	GGG GAC AAG TTT GTA CAA AAA AGC AGG CTA CC ATGGGTTCTCATCATCATCATC
		R-attB2	GGG GAC CAC TTT GTA CAA GAA AGC TGG GTT TCAGGAGAGCACACACTT
XbaI-CAAX-PstI (to make p3E-2A-GCaMP6s-CAAX)	n/a, annealed oligos	F ultramer	CTAGAAAGCTGAACCCTCCTGATGAGAGTGGCCCCGGCTGCATGAGCTGCAAGTGTGTGCTCTCCTGACTGCA
PstI-CAAX-XbaI (to make p3E-2A-GCaMP6s-CAAX)	n/a, annealed oligos	R ultramer	GTCAGGAGAGCACACACTTGCAGCTCATGCAGCCGGGGCCACTCTCATCAGGAGGGTTCAGCTTT

EcoRI-GCaMP6s-XbaI (to make p3E-2A-GCaMP6s-CAAX)	Addgene #40753	F-EcoRI	CCGGAATTCAATGGGTTCTCATCATCATCATC
		R-XbaI	TGCTCTAGACTTCGCTGTCATCATTTGTACA
pME-sypHy	Addgene #24478	F-attB1	GGG GAC AAG TTT GTA CAA AAA AGC AGG CTA CC ATGGACGTGGTGAATCAGCTGG
		R-attB2	GGG GAC CAC TTT GTA CAA GAA AGC TGG GTT CATCTGATTGGAGAAGGAGGTGGG
p3E-mScarlet	Addgene #85042	F-attB2r	GGG GAC AGC TTT CTT GTA CAA AGT GG AA ATGGTGAGCAAGGGCGAGGC
		R-attB3	GGG GAC AAC TTT GTA TAA TAA AGT TG CTTGTACAGCTCGTCCATGCCG
p3E-cadm2a	cDNA	F-attB2r	GGG GAC AGC TTT CTT GTA CAA AGT GG AA ATGATGGTGAAGCAGCATATGC
		R-attb3	GGG GAC AAC TTT GTA TAA TAA AGT TG CTAAATGAAATACTCTTTCTTCTCT
p3E-2A-BoNT/B	pQL86-eGFP-BoNT/B (yeast codon optimized)	F-EcoRI	CCG GAATTC A ATGCCTGTCACCATAAACAACTT
		R-Xba1	TGC TCTAGA CTATTTGACGGATTTGCACATTTG
p3E-2A-dnVamp2 (residues 1-89)	cDNA	F-EcoRI	CCG GAATTC A ATGTCTGCCCCAGCCGGAG
		R-XbaI	TGC TCTAGA CATCTTGGCATTCTTCCACCAGTA
pME-caska	cDNA	F-attB1	GGG GAC AAG TTT GTA CAA AAA AGC AGG CTA CC ATGGCCGACGACGACGTG
		R-attB2	GGG GAC CAC TTT GTA CAA GAA AGC TGG GTT GTAGACCCAGGACACCGGAAC
pDR274-5’cadm1b (to make gRNA synthesis template)	n/a, annealed oligos	F	TAGGATCCAACATGCGCTAGAGAC
		R	AAACGTCTCTAGCGCATGTTGGAT

pDR274-5'dlg4b (to make gRNA synthesis template)	n/a, annealed oligos	F	TAGGTTCCAGGCTAATCACCTGG
		R	AAACCCAGGTGATTAGCCTGGAA
