# Supplementary material for: Oligodendrocytes express synaptic proteins that modulate myelin sheath formation
Source: Nat Commun. 2019 Sep 11;10:4125. doi: 10.1038/s41467-019-12059-y (PMC6739339; doi:10.1038/s41467-019-12059-y)
Supplement: Supplementary file 3 — Reporting Summary [file 41467_2019_12059_MOESM3_ESM.pdf]

nature research

Corresponding author(s): Bruce Appel

Reporting Summary

Nature Research wishes to improve the reproducibility of the work that we publish. This form provides structure for consistency and transparency in reporting. For further information on Nature Research policies, see Authors & Referees and the Editorial Policy Checklist.

Statistical parameters

When statistical analyses are reported, confirm that the following items are present in the relevant location (e.g. figure legend, table legend, main text, or Methods section).

n/a	Confirmed	
☐	☒	The exact sample size (n) for each experimental group/condition, given as a discrete number and unit of measurement
☐	☒	An indication of whether measurements were taken from distinct samples or whether the same sample was measured repeatedly
☐	☒	The statistical test(s) used AND whether they are one- or two-sided<br>*Only common tests should be described solely by name; describe more complex techniques in the Methods section.*
☒	☐	A description of all covariates tested
☐	☒	A description of any assumptions or corrections, such as tests of normality and adjustment for multiple comparisons
☐	☒	A full description of the statistics including central tendency (e.g. means) or other basic estimates (e.g. regression coefficient) AND variation (e.g. standard deviation) or associated estimates of uncertainty (e.g. confidence intervals)
☐	☒	For null hypothesis testing, the test statistic (e.g. F, t, r) with confidence intervals, effect sizes, degrees of freedom and P value noted<br>*Give P values as exact values whenever suitable.*
☒	☐	For Bayesian analysis, information on the choice of priors and Markov chain Monte Carlo settings
☒	☐	For hierarchical and complex designs, identification of the appropriate level for tests and full reporting of outcomes
☐	☒	Estimates of effect sizes (e.g. Cohen's d, Pearson's r), indicating how they were calculated
☐	☒	Clearly defined error bars<br>*State explicitly what error bars represent (e.g. SD, SE, CI)*

Our web collection on statistics for biologists may be useful.

Software and code

Policy information about availability of computer code

Data collection

Provide a description of all commercial, open source and custom code used to collect the data in this study, specifying the version used OR state that no software was used.

Data analysis

Provide a description of all commercial, open source and custom code used to analyse the data in this study, specifying the version used OR state that no software was used.

For manuscripts utilizing custom algorithms or software that are central to the research but not yet described in published literature, software must be made available to editors/reviewers upon request. We strongly encourage code deposition in a community repository (e.g. GitHub). See the Nature Research guidelines for submitting code & software for further information.

Data

Policy information about availability of data

All manuscripts must include a data availability statement. This statement should provide the following information, where applicable:

- Accession codes, unique identifiers, or web links for publicly available datasets
- A list of figures that have associated raw data
- A description of any restrictions on data availability

All analysis was carried out in R. R Markdown files available upon request.

Field-specific reporting

Please select the best fit for your research. If you are not sure, read the appropriate sections before making your selection.

☒ Life sciences ☐ Behavioural & social sciences ☐ Ecological, evolutionary & environmental sciences

For a reference copy of the document with all sections, see nature.com/authors/policies/ReportingSummary-flat.pdf

Life sciences study design

All studies must disclose on these points even when the disclosure is negative.

Sample size	For the dominant-negative work, we predetermined sample size by using mean values for tetanus toxin effect on sheath length published in Hines et al (2015) to determine how many cells/sheaths to use to identify an effect of similar size. Other sample sizes are within range of similar work in the field.
Data exclusions	No data were excluded from analysis.
Replication	All attempts at replication were successful.
Randomization	Randomization was not used for studies on wildtype cells or animals. For dominant-negative work, expression of different DN constructs was performed on wildtype clutchmates in variable order on different injection days. All injections were performed at the 1-cell stage when embryos are indistinguishable from one another.
Blinding	Investigators were blinded during image analysis using a publicly available Fiji plugin (https://github.com/Macklin-Lab/imagej-microscopy-scripts, blind-files_.py)

Reporting for specific materials, systems and methods

Materials & experimental systems

n/a	Involved in the study
☐	☐ Unique biological materials
☒	☐ Antibodies
☒	☐ Eukaryotic cell lines
☒	☐ Palaeontology
☐	☒ Animals and other organisms
☒	☐ Human research participants

Methods

n/a	Involved in the study
☒	☐ ChIP-seq
☒	☐ Flow cytometry
☒	☐ MRI-based neuroimaging

Unique biological materials

Policy information about availability of materials

Obtaining unique materials	All unique materials are readily available upon request.

Animals and other organisms

Policy information about studies involving animals; ARRIVE guidelines recommended for reporting animal research

Laboratory animals	Zebrafish (Danio rerio), AB strain, age 3-8 days post fertilization, were used for all experiments. Animals were all either transgenic or transient-transgenic to label cells of interest and no mutant strains were used.
Wild animals	N/A
Field-collected samples	N/A
